# Hydrogen Sulfide Protects against Chronic Unpredictable Mild Stress-Induced Oxidative Stress in Hippocampus by Upregulation of BDNF-TrkB Pathway

**DOI:** 10.1155/2016/2153745

**Published:** 2016-07-25

**Authors:** Min Hu, Wei Zou, Chun-Yan Wang, Xi Chen, Hui-Ying Tan, Hai-Ying Zeng, Ping Zhang, Hong-Feng Gu, Xiao-Qing Tang

**Affiliations:** ^1^Department of Neurology, Nanhua Affiliated Hospital, University of South China, Hengyang, Hunan 421001, China; ^2^Institute of Neuroscience, Medical College, University of South China, Hengyang, Hunan 421001, China; ^3^Department of Pathophysiology, Medical College, University of South China, Hengyang, Hunan 421001, China; ^4^Department of Anatomy, Medical College, University of South China, Hengyang, Hunan 421001, China

## Abstract

Chronic unpredictable mild stress (CUMS) induces hippocampal oxidative stress. H_2_S functions as a neuroprotectant against oxidative stress in brain. We have previously shown the upregulatory effect of H_2_S on BDNF protein expression in the hippocampus of rats. Therefore, we hypothesized that H_2_S prevents CUMS-generated oxidative stress by upregulation of BDNF-TrkB pathway. We showed that NaHS (0.03 or 0.1 mmol/kg/day) ameliorates the level of hippocampal oxidative stress, including reduced levels of malondialdehyde (MDA) and 4-hydroxy-2-trans-nonenal (4-HNE), as well as increased level of glutathione (GSH) and activity of superoxide dismutase (SOD) in the hippocampus of CUMS-treated rats. We also found that H_2_S upregulated the level of BDNF and p-TrkB protein in the hippocampus of CUMS rats. Furthermore, inhibition of BDNF signaling by K252a, an inhibitor of the BDNF receptor TrkB, blocked the antioxidant effects of H_2_S on CUMS-induced hippocampal oxidative stress. These results reveal the inhibitory role of H_2_S in CUMS-induced hippocampal oxidative stress, which is through upregulation of BDNF/TrkB pathway.

## 1. Introduction

Mood and anxiety disorders have been substantially associated with stressful life. They frequently appear early in life events, cause a chronic course, and adversely affect individual's productive life [1, 2]. Moreover, the current synthetic antistress drugs have low efficacy and even severe adverse-effects. Therefore, understanding the prevention bases of these disorders is crucial. Chronic unpredictable stress (CUMS) is a moderate intensity of stress, which treats experimental animals mainly through long-term and given relatively various mild stressors. Our previous studies reported that CUMS results in damage to the hippocampus [3]. Notably, it has been shown that stress can lead to neuronal atrophy and loss in certain brain structures, mainly in the hippocampus [1, 4]. In parallel, exogenous stress is also reported to induce neuronal cell death in the hippocampus [5]. Meanwhile, elevated hippocampal oxidative stress plays a vital role for neurotoxicity and neuronal death toward the progression of CUMS-treated rats [6, 7].

Hydrogen sulfide (H_2_S), the third gaseous mediator [8], has been recognized to play crucial important roles in physiological functions of central nervous system [9, 10]. H_2_S enhances the induction of long-term potentiation (LTP) in the hippocampus [11, 12] and regulates intracellular Ca^2+^ waves in neurons [13, 14], which indicates that H_2_S is a neuromodulator. Interesting, the critical role of H_2_S in suppressing oxidative stress has been confirmed [15, 16]. In addition, recent report demonstrated that exogenous H_2_S alleviated the oxidative stress-induced rat hippocampal damage through its antioxidant effects [17]. Moreover, we have previously provided compelling evidence that CUMS induced the imbalance of proportion to endogenous H_2_S in hippocampus [18]. Thus, we speculated that H_2_S attenuates oxidative stress in hippocampus of CUMS-treated rats.

Brain-derived neurotrophins factor (BDNF) is a member of the neurotrophins family, which exerts its roles via its high affinity receptor tyrosine protein kinase B (TrkB) [19]. BDNF and its receptor TrkB, which are widely and abundantly expressed throughout in the CNS, activate various intracellular signaling pathways associated with the neuroprotective effects [20]. Numerous studies have also documented that stress significantly decreases BDNF mRNA expression in the hippocampus [4, 21]. Recent studies reported that stress decreases the expression of BDNF in the frontal cortex and hippocampus of rodents [22]. Meanwhile, it is conceivable that BDNF downregulates the ethanol-induced cellular oxidative stress and apoptosis in developing hypothalamic neuronal cells [23]. Furthermore, our previous studies have shown that BDNF-TrkB pathway mediates the protective role of H_2_S against FA-induced oxidative damage in PC12 cells [24]. Therefore, we will investigate whether the protection of H_2_S against CUMS-induced hippocampal oxidative stress is also via BDNF/TrkB pathway.

In the current study, our results identified the suppressive effects of H_2_S on hippocampal oxidative stress in CUMS-exposed rats. We also demonstrated that H_2_S significantly rescues the downregulation of BDNF expression in the hippocampus of CUMS-exposed rats. Meanwhile, K252a, a BDNF-TrkB pathway inhibitor, abolished the protective effects of H_2_S against CUMS-induced oxidative stress. Taken together, we identified a critical role of H_2_S in protection against CUMS-induced oxidative stress in hippocampus, as a result of upregulation of BDNF-TrkB pathway.

## 2. Materials and Methods

### 2.1. Animals

Adult male Sprague-Dawley (SD) rats (250–280 g) were purchased from the Hunan SJA Laboratory Animal Center (Changsha, Hunan, China). Rats were housed individually and given free access to food and water under a normal 12 h light/dark schedule (lights on at 07:00 a.m.). Room temperature was maintained at 22 ± 1°C and relative humidity of 55% ± 5%. Rats were allowed 7 days to acclimatize themselves to the housing conditions before the beginning of the experiments. All the procedures were strictly conducted according to the National Institutes of Health Guide for the Care and Use of Laboratory Animals and were approved by the Animal Use and Protection Committee of University of South China.

### 2.2. Reagents

Sodium hydrosulfide (NaHS, a donor of H_2_S) and K252a (a selective pharmacological p-TrkB inhibitor) were purchased from Sigma (Sigma, St. Louis, MO, USA). Specific monoclonal antibodies for detecting BDNF and p-TrkB were obtained from Epitomic Inc. (Burlingame, UK). *β*-actin antibody and goat anti-rat antibody were purchased from Proteintech (Danvers, MA, USA). The 4-hydroxy-2-trans-nonenal (4-HNE) assay kit, malondialdehyde (MDA) assay kit, superoxide dismutase (SOD) assay kit, and glutathione (GSH) enzyme-linked immunosorbent assay (ELISA) kits were bought from USCN Life Science Inc. (Wuhan, Hubei, China). Bicinchoninic Acid (BCA) Protein Assay Kit was obtained from Beyotime Institute of Biotechnology (Shanghai, China).

### 2.3. Lateral Ventricle Cannulation

Rats were first anesthetized with sodium pentobarbital (60 mg/kg, i.p., Sigma, St. Louis, MO, USA) and then placed in stereotaxic apparatus for operation; a permanent guide cannula was aseptically and stereotaxically implanted into the right lateral ventricle of rats using the coordinates (AP, −1.0 mm; ML, +2 mm; DV, +3.5 mm). After stereotaxic surgery, home-cage behavior and wound healing were monitored. Body weight recovery to no less than 90% of presurgery amount within 7 days was the inclusion criteria.

### 2.4. CUMS Procedure

Cannulated rats were exposed to a series of variable stressors per day for 2 weeks as described previously with minor modifications [25]. Briefly, animals were exposed to the series stress regime consisting of 24 h food deprivation, 24 h water deprivation, exposure to an empty bottle for 1 h, 7 h cage tilt (45°), overnight illumination, 24 h soiled cage (200 mL of water in 100-g sawdust bedding), 30 min forced swimming at 8°C, 3 h physical restraint, and 24 h exposure to a unfamiliar object (e.g., a piece of plastic). All of these stressor episodes were randomly scheduled over a 1-week period and repeated throughout the 4-week experiment.

### 2.5. Drug Administration

CUMS rats were exposed to one stressor per day for 2 weeks and then received 2 weeks of NaHS (0.03 or 0.1 mmol/kg/day) or saline intraperitoneal injections or K252a (1 *μ*g) intracerebroventricularly (i.c.v.) during continuous CUMS treatment. Control rats were left undisturbed in their home cages with the exception of general handling for 2 weeks and intraperitoneally injected with saline in the last 2 weeks.

### 2.6. Novelty-Suppressed Feeding Test

The Novelty-suppressed feeding test (NSFT) was performed according to previous protocols [26], with a minor modification. Rats were initially food deprived for 24 h in their home cages and then transferred to a new chamber without habituation. Regular food pellets (weighed) were placed in the center of the testing room. Each subject was first placed in a corner of the testing area. The latency to begin to chew the pellet was recorded. And then rats were transferred to their home cage; the amount of food consumed for 30 min was measured.

### 2.7. Elevated Plus-Maze Test

Anxiolytic activity was measured in the elevated plus-maze (EPM) test [27]. In this test, the maze (EPMR; Shanghai Jiliang Software Technology Co. Ltd, Shanghai, China) consisted of four crossed narrow arms (50 cm long, 10 cm wide), with two open arms and two closed arms (40 cm high). The apparatus was elevated 70 cm from the floor. During the EPM test, rats were individually first placed in the central zone of the EPM, head facing an open arm, and the rat were allowed to freely explore the maze for 5 min. The total number of arm entries and the percentage of entries into and the proportion of time spent in the open arms were assessed.

### 2.8. Biochemical Analysis for MDA, 4-HNE, SOD, and GSH

The hippocampal tissues were homogenized (10% w/v) with 0.1 mol/L of PBS and centrifuged at 12,000 g for 10 min. The supernatants were collected and the total protein concentration was quantified by BCA Protein Assay. The levels of MDA, 4-HNE, and GSH and the activity of SOD in the supernatant of hippocampus were, respectively, measured by enzyme-linked immunosorbent assay (ELISA) under standard conditions, according to the manufacturer's instructions on the reagent kits. The absorbance was read at 450 nm using a microplate reader.

### 2.9. Western Blot Analysis for the Levels of BDNF and p-TrkB Protein Expression

Protein from hippocampal tissue was homogenized with ice-cold homogenizing buffer and incubated on ice for 30 min. The homogenates were centrifuged at 14,000 g for 10 min at 4°C. The total protein of supernatant was subsequently measured by using BCA assay kit (Beyotime, Shanghai, China). Equal amounts of total protein (25 *μ*g/lane) were separated on 8–12% SDS-PAGE and electrotransferred to PVDF membranes. After blocking the membranes with TBST containing 5% nonfat dried milk for at least 2 h at room temperature, membranes were incubated with the primary antibody (anti-BDNF, 1 : 1000, Epitomics; anti-p-TrkB, 1 : 1000, Epitomics; *β*-actin 1 : 2000, Proteintech) at 4°C overnight. Then the blots were washed three times for 8 min by using TBST and incubated with secondary antibody for 1 h. After washing, protein bands were analyzed using the enhanced chemiluminescence detection system (BeyoECLPlus kit, Beyotime, P0018). The signal of the immunoblot was analyzed using Image J software and calculated with *β*-actin (a loading control).

### 2.10. Statistical Analysis

All data are expressed as mean ± SEM. The significance of intergroup differences was evaluated by one-way analyses of variance, followed by LSD* post hoc* tests. A value of *P* < 0.05 was considered significant.

## 3. Results

### 3.1. H_2_S Decreases the Levels of MDA and 4-HNE in the Hippocampus of CUMS-Exposed Rats

**Figure 1:**
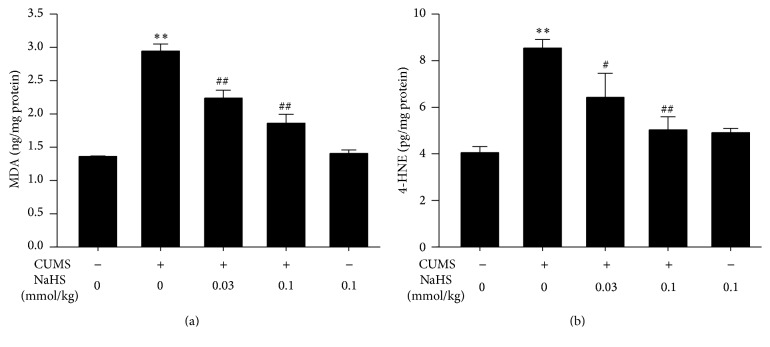
Effect of H_2_S on the generations of MDA and 4-HNE in the hippocampus of CUMS-exposed rats. Rats were exposed to CUMS for 2 w and then cotreated with NaHS (0.03 or 0.1 mmol/kg/day, i.p.) and CUMS for 2 w. The levels of MDA (a) and 4-HNE (b) in the hippocampus of rats were detected by ELISA kit. Values are expressed as mean ± SEM (*n* = 3–5/group). ^*∗∗*^ *P* < 0.01, versus the control group; ^#^ *P* < 0.05, ^##^ *P* < 0.01, versus the CUMS- treated group.

MDA and 4-HNE are the markers of lipid peroxidation to indicate the oxidative stress level. There was a significant decrease in the levels of MDA (Figure 1(a)) and 4-HNE (Figure 1(b)) in the hippocampus of CUMS-exposed rats as compared with those of control rats. However, treatment with NaHS (a donor of H_2_S, 0.03 or 0.1 mmol/kg/day, i.p., for 2 w) significantly reduced the levels of MDA (Figure 1(a)) and 4-HNE (Figure 1(b)) in the hippocampus of CUMS-exposed rats. These data indicated that H_2_S prevents CUMS-induced hippocampal oxidative stress.

### 3.2. H_2_S Increases the SOD Activity and GSH Level in the Hippocampus of CUMS-Exposed Rats

**Figure 2:**
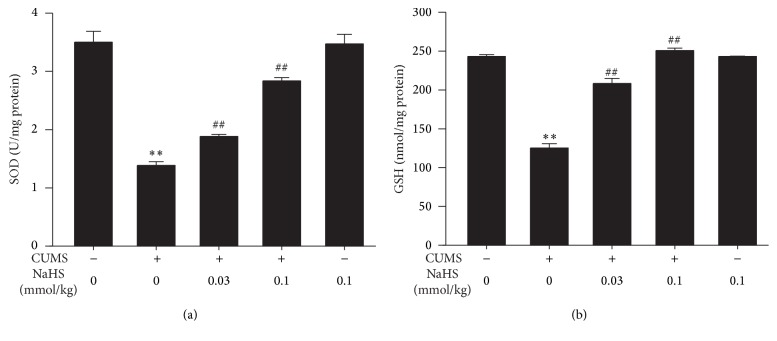
Effect of H_2_S on the SOD activity and GSH level in the hippocampus of CUMS-exposed rats. Rats were exposed to CUMS for 2 w and then cotreated with NaHS (0.03 or 0.1 mmol/kg/day, i.p.) and CUMS for 2 w. The SOD activity (a) and GSH level (b) in the hippocampus of rats were detected by ELISA kit. Values are expressed as mean ± SEM (*n* = 3–5/group). ^*∗∗*^ *P* < 0.01, versus the control group, ^##^ *P* < 0.01, versus the CUMS-treated alone group.

We also investigated the effects of H_2_S on the SOD activity and GSH level in the hippocampus of CUMS-treated rats. The SOD activity (Figure 2(a)) and GSH level (Figure 2(b)) were significantly reduced in the hippocampus of CUMS-treated rats. However, treatment with NaHS (0.03 or 0.1 mmol/kg/day, i.p., for 2 w) increased the SOD activity and GSH level in the hippocampus of CUMS-treated rats, which further indicated the protective action of H_2_S against CUMS-exerted hippocampal oxidative stress.

### 3.3. H_2_S Upregulates the Level of BDNF and p-TrkB Protein in the Hippocampus of CUMS-Exposed Rats

**Figure 3:**
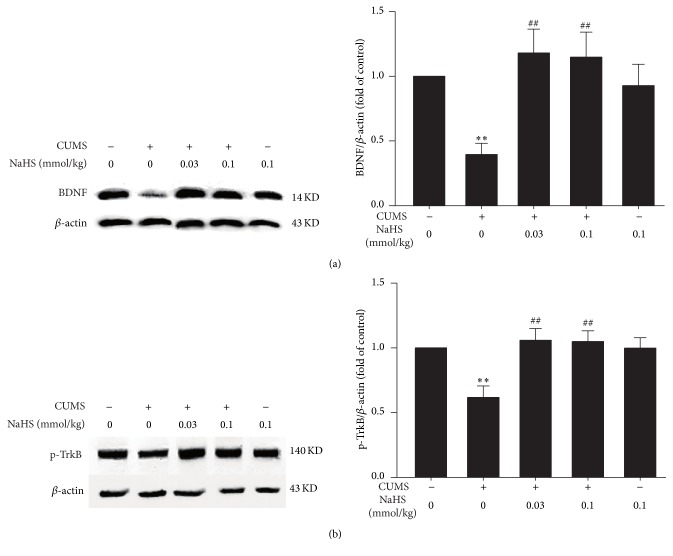
Effect of H_2_S on the expressions of BDNF and p-TrkB protein in the hippocampus of CUMS-treated rat. Rats were exposed to CUMS for 2 w and then cotreated with NaHS (0.03 or 0.1 mmol/kg/day, i.p.) and CUMS for 2 w. Immunoblot was used to analyze and quantify the expressions of BDNF and p-TrkB in the hippocampus of rats, and *β*-actin was used as an internal control. Values were expressed as the mean ± SEM of three independent experiments. ^*∗∗*^ *P* < 0.01, versus control group; ^##^ *P* < 0.01, versus CUMS-treated along group.

To determine whether BDNF/TrkB pathway is involved in the protective effect of H_2_S against CUMS-induced hippocampal oxidative stress, we first explored the effects of H_2_S on the expression of BDNF and p-TrkB protein in CUMS-exposed rats. After 4 w exposure of CUMS, the expressions of BDNF (Figure 3(a)) and p-TrkB (Figure 3(b)) protein in the hippocampus were significantly decreased in the hippocampus of rats. However, after treatment with NaHS (0.03 or 0.1 mmol/kg/day, i.p., for 2 w), the expressions of BDNF (Figure 3(a)) and p-TrkB (Figure 3(b)) proteins in the hippocampus of CUMS-exposed rats were markedly increased, which indicated that H_2_S upregulates the hippocampal BDNF-TrkB pathway in CUMS-exposed rats.

### 3.4. K252a Reverses the Inhibitory Role of H_2_S in CUMS-Increased MDA and 4-HNE in the Hippocampus

**Figure 4:**
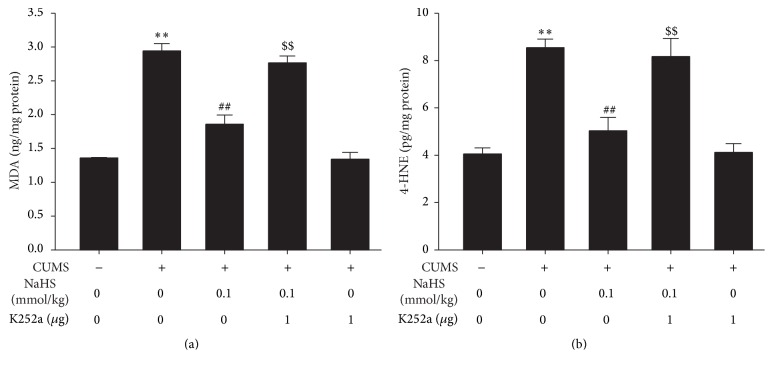
Effect of k252a on H_2_S-caused decrease in MDA and 4-HNE generation in the hippocampus of CUMS rats. The levels of MDA (a) and 4-HNE (b) in the hippocampus of rats were detected by ELISA kit. Values are expressed as mean ± SEM (*n* = 5-6/group). ^*∗∗*^ *P* < 0.01, versus control group; ^##^ *P* < 0.01, versus CUMS-treated alone group; ^$$^ *P* < 0.01, versus CUMS and H_2_S-treated group.

To confirm the mediatory role of BDNF-TrkB pathway in the protection of H_2_S against CUMS-induced hippocampal oxidative stress, we further explored whether K252a, a specific BDNF-TrkB pathway inhibitor, reverses the protective role of H_2_S against hippocampal oxidative stress in CUMS-exposed rats. As shown in Figure 4, K252a (1 *μ*g, i.c.v.) eliminated the protection of NaHS (0.1 mmol/kg, i.p.) against CUMS-induced increases in hippocampal MDA (Figure 4(a)) and 4-HNE (Figure 4(b)) levels, while treatment with K252a (1 *μ*g) alone did not affect the MDA (Figure 4(a)) and 4-HNE (Figure 4(b)) levels in control rats, which indicated that K252a blocks the protection of H_2_S against hippocampal oxidative stress in CUMS-exposed rats.

### 3.5. K252a Reverses the Upregulatory Role of H_2_S in the SOD Activity and GSH Level in the Hippocampus of CUMS-Exposed Rats

**Figure 5:**
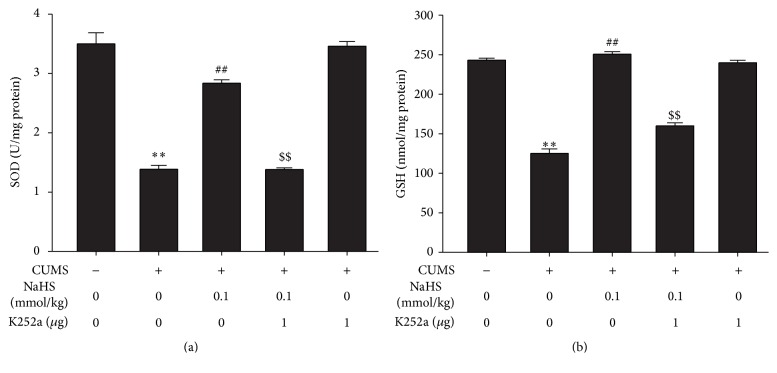
Effect of k252a on H_2_S upregulated SOD activity and GSH level in the hippocampus of CUMS-exposed rats. The SOD activity (a) and GSH level (b) in the hippocampus of rats were detected by ELISA kit. Values are expressed as mean ± SEM (*n* = 5-6/group). ^*∗∗*^ *P* < 0.01, versus control group; ^##^ *P* < 0.01, versus CUMS-treated alone group; ^$$^ *P* < 0.01, versus CUMS and H_2_S-treated group.

To further determine the mediatory role of BDNF-TrkB pathway in the protection of H_2_S against CUMS-induced hippocampal oxidative stress, we also explored whether K252a blocks the upregulatory role of H_2_S in the SOD activity and GSH level. K252a (1 *μ*g, i.c.v.) significantly decreased the SOD activity (Figure 5(a)) and GSH level (Figure 5(b)) in the hippocampus of NaHS (0.1 mmol/kg, i.p.) and CUMS cotreated rats. This result also suggests that BDNF/TrkB pathway is involved in the protection of H_2_S against CUMS-induced hippocampal oxidative stress.

### 3.6. H_2_S Exerts Antidepressive- and Anxiolytic-Like Effects on CUMS-Exposed Rats

**Figure 6:**
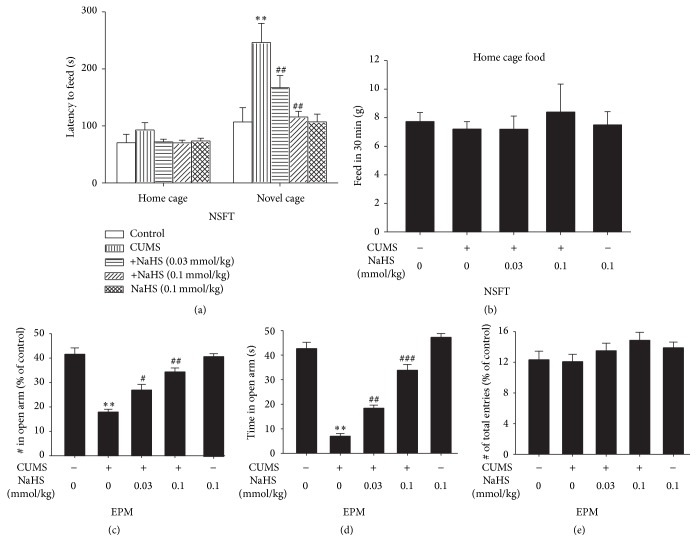
Effect of H_2_S on depressive- and anxiety-like behaviors in CUMS-exposed rats. Rats were exposed to CUMS for 2 w and then cotreated with NaHS (0.03 or 0.1 mmol/kg/day, i.p.) and CUMS for 2 w. (a)-(b) The depressive-like behavior was evaluated using novelty-suppressed feeding test (NSFT). (c)–(e) The anxiety-like behavior was evaluated using elevated plus-maze (EPM) test. ^*∗∗*^ *P* < 0.01 versus control group, ^##^ *P* < 0.01, versus CUMS-treated alone group. ^###^ *P* < 0.001, versus CUMS-treated group. Data represent group mean ± SEM (*n* = 7–10/group).

We further verified the ability of H_2_S to prevent CUMS-induced depression- and anxiety-like behaviors. The NSF test has been suggested to have high predictive validity for antianxiety activity [26]. As shown in Figure 6(a), CUMS-exposed rats displayed depression-like behaviors reflected by an increase in the time of latency to feed; however, treatment with NaHS (0.03 or 0.1 mmol/kg/day, i.p., for 2 w) decreases the latency to feed in the CUMS-exposed rats, reflecting the antidepressive-like action of H_2_S. NaHS treatment did not affect the food consumption at home cage during the 30 min test session (Figure 6(b)), indicating that the antidepressive-like effect of NaHS was not due to the possibility that NaHS affected the normal appetite and feeding. To verify the possibility that H_2_S exerts an anxiolytic-like activity in CUMS rats, rats were subjected to the EPM test. After they were treated with NaHS (0.03 or 0.1 mmol/kg/day, intraperitoneally) for 2 w, we found an increase in the proportion of entries into (Figure 3(c)) and the time spent in (Figure 3(d)) the open arms, without significant change in total arm entries (Figure 3(e)) compared to the saline-treated CUMS rats, indicating an anxiolytic-like effect of H_2_S in CUMS rats.

## 4. Discussion

H_2_S, a novel endogenous gaseous mediator, has been recognized as an antioxidant [28, 29] and our group has demonstrated the decrease of endogenous H_2_S in the hippocampus of CUMS-exposed rats [18]. Emerging evidence supports that brain-derived neurotrophic factor (BDNF) has neuroprotective effect. In the present work, we investigated whether H_2_S inhibits CUMS-induced hippocampal oxidative stress and whether BDNF/TrkB pathway mediates this inhibitory role of H_2_S. We found that H_2_S decreased oxidative stress and increased the expressions of BDNF and p-TrkB in the hippocampus of CUMS-exposed rats. Moreover, this protection effect of H_2_S against oxidative stress was abolished by K252a, an inhibitor of TrkB. This is the first report demonstrating the protection of H_2_S against CUMS-induced hippocampal oxidative stress, as a result of upregulation of BDNF-TrkB pathway.

Oxidative stress contributes to the pathophysiologic cascade relating with hippocampus injury [30]. Similarly, cumulative evidence indicates that oxidative stress in the hippocampus exerts a vital role in CUMS-induced pathogenesis of rodents [31–37]. Lowered antioxidant enzyme activity within the central nervous system may impair protection against reactive oxygen species (ROS), leading to damage to intracellular structure such as DNA, fatty acid, and protein [38–41]. Recently, the potential antioxidant and cytoprotective effects of H_2_S have been a focus of research [42]. It has shown that H_2_S scavenges oxidants and reactive aldehydes, such as 4-HNE, MDA, and peroxynitrite, by modulating the GSH content and SOD activity [43–46]. Thus, we investigated whether H_2_S protects hippocampus against CUMS-induced oxidative stress. In our study, CUMS-treated rats exerted a significant increase in the concentration of lipid peroxidation, MDA, and 4-HNE and marked reduction in the GSH level and SOD activity of the hippocampus. However, these parameters were significantly reversed by NaHS (a donor of H_2_S) administration. NaHS notably decreased the levels of MDA and 4-HNE and increased the GSH level and SOD activity in the hippocampus of CUMS-treated rats. These data suggested that H_2_S attenuates oxidative stress in the hippocampus of CUMS-exposed rats. Considering that CUMS is well established to cause depressive- and anxiety-like behaviors, it would be important to verify the ability of H_2_S to abolish CUMS-induced depressive- and anxiety-like behaviors. In the present work, we found that H_2_S prevented CUMS-caused depressive-like behavior in the NSF test and anxiety-like behavior in the EPM test, which are consistent with our previous study [47].

BDNF modulates cellular function by activation of its specific tyrosine kinase receptor, TrkB. Numerous studies have also documented that BDNF has neuroprotective effects [48–51]. Interestingly, our previous study confirmed that H_2_S increases the expression of BDNF protein in the hippocampus of rats [52]. BDNF also possesses antioxidant activities [53]. Meanwhile, our data are consistent with previously reported data that CUMS decreases BDNF levels in the hippocampus [34]. We also found that treatment of rat with NaHS intraperitoneal injections increased the expression of BDNF and p-TrkB proteins in the hippocampus of CUMS-exposed rats. Therefore, we speculate whether the BDNF-TrkB pathway mediates the protection of H_2_S against oxidative stress in the hippocampus of CUMS-treated rats. We infused K252a, the TrkB receptor antagonist, to inhibit BDNF-TrkB signaling in the brain. Subsequently, we detected the two endogenous aldehydes levels of MDA and HNE, which are commonly used as a marker of oxidative stress [54, 55]. We found that K252a blunt H_2_S antioxidant effect in CUMS-exposed rats. In line with this, the inhibitory role of H_2_S in CUMS-increased MDA and 4-HNE was reversed by K252a in the hippocampus of CUMS-treated rats. In addition, K252a was able to completely block the upregulatory role of H_2_S in the SOD activity and GSH level in the hippocampus of CUMS-exposed rats. Collectively, these findings suggest that BDNF/TrkB pathway mediates the protection of H_2_S against CUMS-induced hippocampal oxidative stress.

Finally, our data suggest that H_2_S protects against CUMS-induced hippocampal oxidative stress by modulating BDNF/TrkB singling. It is still unclear whether H_2_S-derived bioactive molecules will be involved in regulating hippocampal oxidative system. Interestingly, recent study shows that endogenous diffusible polysulfides (H_2_S_*n*_) in rat brain are released from H_2_S [56, 57]. Further experimental and clinical studies are still needed to determine the antioxidant activity of H_2_S.

In conclusion, the present study demonstrated that H_2_S inhibits CUMS-induced hippocampal oxidative stress and upregulates the hippocampal BDNF-TrkB pathway in CUMS-exposed rats and that the blockage of BDNF-TrkB pathway reverses the protection of H_2_S against CUMS-induced oxidative stress. Our data establish a key role of H_2_S against CUMS-induced hippocampal oxidative stress through BDNF-TrkB pathway and provide a basis for investigating H_2_S as a therapeutic approach for the pathogenesis of stress.
